# Transarterial chemoembolization combined with sorafenib for advanced hepatocellular carcinoma

**DOI:** 10.3892/ol.2014.2512

**Published:** 2014-09-09

**Authors:** WENBO SHAO, FENGJUAN ZHANG, NING CONG, JINPENG LI, JINLONG SONG

**Affiliations:** Department of Surgical Oncology (Interventional Therapy), Shandong Cancer Hospital and Institute, Shandong Academy of Medical Sciences, Jinan, Shandong 250117, P.R. China

**Keywords:** sorafenib, tansarterial chemoembolization, hepatocellular carcinoma

## Abstract

Sorafenib has been demonstrated to improve survival rate in patients with advanced hepatocellular carcinoma (HCC); however, the survival benefit remains modest and the response rates remain poor. Transarterial chemoembolization (TACE) may be used for the treatment of advanced HCC with well-preserved liver function and has a high local tumor control rate. We hypothesized that patients with advanced HCC may benefit from the combination of sorafenib with TACE. A retrospective study was conducted involving patients with advanced HCC, who had received at least one TACE session. Patients subsequently received 400 mg sorafenib twice per day and were monitored monthly. A dose reduction from 400 to 200 mg of sorafenib twice per day was permitted. The overall survival and side effects were subsequently followed up. In total, 38 patients were included from April 1st, 2009 to March 31st, 2012. All patients were treated with sorafenib after TACE was performed. As of March 31st, 2013, seven patients remained on sorafenib, and were censored at that time point. The median overall survival time was 12 months (95% confidence interval, 6.3–17.7 months). The sorafenib dose was reduced temporarily in 32 patients (84.2%). The most common toxicities were dermatological adverse effects (94.7%), diarrhea (63.2%) and alopecia (26.3%). The survival benefit of sorafenib combined with TACE for advanced HCC is promising, with no intolerable adverse events, provided that dose adjustment is permitted.

## Introduction

Hepatocellular carcinoma (HCC) is the fifth most common type of cancer and the third leading cause of cancer-related mortality worldwide (1). The majority of cases arise in Asia and Africa, particularly in China (2). Despite the extensive application of intensive surveillance programs implemented over the past few years, numerous patients are not diagnosed until the disease is at an advanced stage, such that only palliative treatment options are available.

Sorafenib, an orally active multikinase inhibitor, is recommend as the standard treatment for advanced HCC [Barcelona clinic liver cancer (BCLC) stage C] in western countries (3). A randomized controlled trial has confirmed that sorafenib can prolong the median overall survival (OS) compared with placebo in patients with advanced HCC (4). A further study confirmed the benefit of sorafenib for patients from the Asia-Pacific region (5). However, the survival among patients with advanced HCC remained modest, and the local tumor control rate was low.

Transarterial chemoembolization (TACE) is the standard treatment for intermediate-stage HCC (3). This procedure has a high local tumor control rate and has been observed to enhance survival in patients with intermediate HCC (6–8). However, the hypoxia caused by TACE in surviving tumor cells leads to the release of angiogenic growth factors, which contribute to tumor recurrence or metastases and a poorer outcome (9,10). Sorafenib blocks tumor cell proliferation by targeting Raf/MEK/ERK signaling at the Raf kinase level, and exerts an antiangiogenic effect by targeting vascular endothelial growth factor receptor-2 and -3, and platelet-derived growth factor receptor-β tyrosine kinases (11). Therefore, the combination of TACE with sorafenib may provide a benefit for patients with HCC. A number of studies have reported that TACE combined with sorafenib significantly prolonged the median OS time or time to progression (TTP) for patients with unresectable HCC (12–14). The majority of the patients included in these studies had intermediate-stage HCC. TACE may also be used in advanced HCC with conserved liver function (15). To date, limited data has focused on the combination of TACE with sorafenib for advanced hepatocellular carcinoma. The aim of the current study was to investigate the OS and side effects of the combination therapy with TACE and sorafenib in patients with advanced HCC.

## Patients and methods

### Patient characteristics

This retrospective study was approved by the Institutional Review Board of Shandong Cancer Hospital and Institute (Jinan, China). A review of patients with advanced HCC treated with TACE prior to sorafenib administration in Shandong Cancer Hospital and Institute was undertaken. All patients were diagnosed by histology, cytology or persistently elevated serum α-fetoprotein (AFP) levels (>400 ng/ml; normal range, 0–7 ng/ml) with typical imaging findings. All patients were staged according to the BCLC staging classification (3). All patients had been previously treated with at least one TACE session prior to sorafenib administration. Written informed consent was obatined from all patients.

### Transarterial chemoembolization

TACE was performed according to the traditional method (16). Chemotherapeutic agents, 100–200 mg oxaliplatin and 750–1,000 mg fluorouracil glycosides, were infused into feeding arteries of the tumor; subsequently, 5–20 ml Lipiodol mixed with 30 mg epirubucin was infused into the feeding arteries at a rate of 1 ml/min until stasis flow in the tumor vascularity was achieved. The use of a gelatin sponge was not required, as determined by the interventional radiologist.

### Sorafenib treatment

Sorafenib was administered 7–30 days following the final TACE treatment. Patients received 400 mg sorafenib twice daily at the beginning of treatment. A dose reduction from 400 to 200 mg of sorafenib twice daily was permitted when drug-related adverse events were observed. When the adverse events subsided, the decision to resume treatment with 400 mg sorafenib twice daily was discussed between the patient and the physician, but was ultimately made by the physician. On the observation of progressive disease, the decision to continue treatment was discussed between the patient and the physician, but was ultimately made by the patient. If treatment was continued, informed consent was required, and treatment with sorafenib would be maintained until a deterioration in the Child-Pugh score to C or ECOG performance status (PS) score to 4 was observed, or until the occurrence of intolerable adverse events or mortality.

### Follow-up

All patients were monitored monthly for the occurrence of side effects, ECOG PS classification and Child-Pugh score evaluation. The follow-up of survival was discontinued on March 31, 2013, and patients that remained alive were censored at that time point. The side effects of sorafenib were reported according to the National Cancer Institute’s Common Terminology Criteria for Adverse Events (version 3.0) (17).

### Statistical analysis

The OS refers to the time between the first administration of sorafenib to mortality by any cause or the final follow-up. Survival analysis was estimated by the Kaplan-Meier survival method. The Cox proportional-hazards regression model was used to assess factors that were independently prognostic of OS. Statistical analysis was performed with SPSS version 16.0 software (SPSS Inc., Chicago, IL, USA).

## Results

### Characteristics of patients and disease

In total, 38 patients were included in the study between April 1st, 2009 and March 31st, 2012. The characteristics of the 38 patients and their diseases including age, gender, ECOG PS, Child-Pugh score and tumor size, as well as the presence of portal vein thrombosis and/or metastasis are summarized in Table I.

### Survival

**Figure 1:**
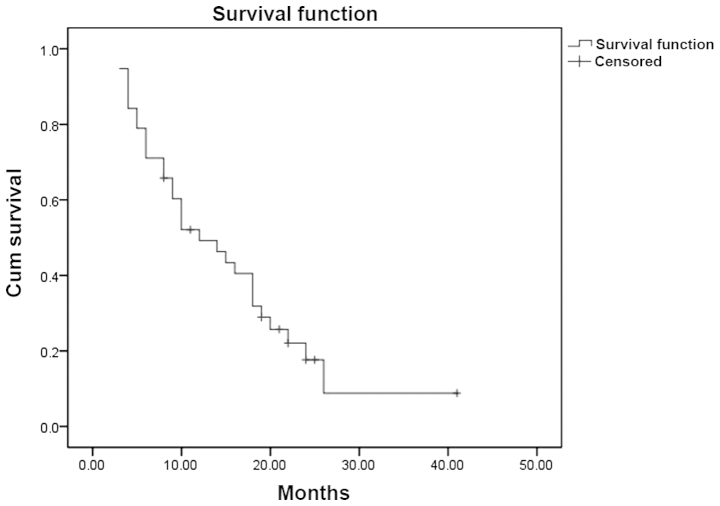
Overall survival times of patients receiving sorafenib following TACE (n=38). The overall median survival time was 12 months.

All 38 patients were subjected to follow-up. Seven patients remained on sorafenib on March 31st, 2013, and were censored at that time point. The median OS time was 12 months (95% confidence interval, 6.3–17.7 months) (Fig. 1). Compared with extrahepatic spread, the Cox proportional-hazards regression model indicated that portal vascular invasion was independently prognostic of OS.

### Side effects

All patients experienced various toxicities at the end of the first month of sorafenib treatment. The most common toxicities were dermatological adverse effects (94.7%), diarrhea (63.2%) and alopecia (26.3%). No grade 3 or 4 adverse events were observed. At the end of month two, the sorafenib dose was reduced to 200 mg twice daily in 32 patients due to toxicity. By the end of month four, the majority of toxicities had been relieved following suitable treatment and dose adjustment. Two patients resumed treatment with 400 mg sorafenib twice daily and no repeated aggravation of the toxicities was observed; however, the majority of patients had to balance the repeated adverse effects and the dose adjustment of sorafenib.

## Discussion

TACE is the standard treatment for intermediate-stage HCC according to the BCLC staging classification (3). However, in Asia, the role of TACE is further extended to include the treatment of advanced HCC. The current study demonstrated that the combination treatment with TACE and sorafenib led to a median OS time of 12 months for hepatitis B virus-related advanced HCC. Compared with the OS time of the Sorafenib Asia-Pacific (6.5 months) and SHARP trials (10.7 months), which used sorafenib monotherapy for untreated advanced HCC (4,5), the survival benefit of sorafenib in the current study is promising. This positive result may be due to TACE treatment reducing the tumor burden by blocking angiogenesis and killing tumor cells through the anticancer agents present, prior to sorafenib administration. Repeated TACE may improve local tumor control, however, it may also worsen liver function; therefore, further treatment with TACE must be avoided if no hypervascularity is observed. In the current study, the mean number of TACE sessions prior to and following sorafenib administration were 4.3 and 0.9, respectively. The reduced frequency of TACE following, as compared with prior to, sorafenib administration, may be due to the antiangiogenic effects of sorafenib.

With regard to safety, the current study demonstrated that sorafenib administration following TACE could be tolerated in patients with advanced HCC, provided that dose adjustment was permitted. The adverse events were deemed to be predominantly sorafenib-related, as sorafenib was administered following the completion of the TACE session. This was in concordance with the results observed in western patients. However, on comparison, the profile of adverse events differed to those reported in western patients. In the study by Pawlick *et al* (18), the most common toxicities were fatigue (94%), anorexia (67%), alteration in liver enzymes (64%) and dermatological adverse effects (48%), whilst in the current study, the most common toxicities were dermatological adverse effects (94.7%), diarrhea (63.2%) and alopecia (26.3%). %). Fewer patients complained of fatigue and more hand-foot skin reactions occurred in the present study. Additionally, no grade 3 or 4 toxicities were identified in our study, which may have been due to the high-rate of sorafenib dose reduction. In the current study, 84.2% of patients reduced the dose of sorafenib in month two, while Dufour *et al* (19) reported that the dose of sorafenib was reduced in only 21.4% of patients. The majority of toxicities were relieved following the dose reduction and were not aggravated further, even the dose of sorafenib was increased to 400 mg twice daily. This also indicated that sorafenib was well tolerated in patients with HCC following TACE treatment.

One aspect of the current study that must be considered is that all patients were treated with sorafenib, despite radiographic progression, until a deterioration was observed in the patient Child-Pugh score to C or the ECOG PS score to 4, or until the occurrence of intolerable adverse events or mortality. This differs from the study by Sansonno *et al* (14), in which sorafenib administration was discontinued in the instance of disease progression. With informed consent, sorafenib treatment was continued in the current study following disease progression, due to the lack of secondary treatment options for advanced HCC and the assumed survival benefit. However, it is uncertain whether the benefit of sorafenib remains following disease progression; therefore, further investigation is required. Furthermore, for advanced HCC patients treated with sorafenib following TACE, portal vascular invasion was identified as an independent risk factor, while extrahepatic spread was not an independent risk factor. This implies that for advanced HCC, patients who do not exhibit portal vascular invasion would be more likely to benefit from sorafenib as adjuvant therapy following TACE.

Three strategies have been identified for the combination of sorafenib with TACE: The sequential approach, the interrupted approach and the continuous approach. Certain studies have reported that TACE combined with sorafenib in an interrupted or continuous approach significantly prolonged the median OS time (12,13). However, the outcome of the sequential approach for sorafenib combined with TACE is controversial. Kudo *et al* (20) reported that sorafenib did not significantly prolong TTP or OS in patients with unresectable HCC who responded to TACE. Conversely, Sansonno *et al* (14) reported that a conventional TACE procedure followed by sorafenib treatment resulted in a significantly longer TTP in patients with intermediate-stage hepatitis C virus-related HCC. In the sequential approach, sorafenib is used as an adjuvant therapy when the TACE sessions have been completed. The results from the current study indicate that sorafenib as an adjuvant therapy following TACE holds promise as a useful strategy for the treatment of patients with advanced HCC.

In conclusion, treatment with sorafenib combined with TACE exhibited a median survival time of 12 months in patients with advanced HCC. The Cox proportional-hazards regression model indicated that portal vascular invasion was independently prognostic of OS. The survival benefit of sorafenib combined with TACE for advanced HCC is promising, with no intolerable adverse events, providing that dose adjustment is permitted.

## Figures and Tables

**Table I:** Baseline characteristics of the study population.

Patient characteristics	n	%
Age, years^a^	53.4 (35–68)	
Gender		
Male	37	97.4
Female	1	2.6
ECOG performance status		
0	36	94.7
1	2	5.3
Child-Pugh score		
A	32	84.2
B	6	15.8
Etiology		
Hepatisis B virus	38	100.0
BCLC status		
C	38	100.0
Diameter of main tumor nodule, cm^a^	5.6 (3–10)	
Portal vein thrombosis	26	68.4
Distant metastasis	20	52.6
Sessions of TACE prior to sorafenib^a^	4.3 (1–7)	
Sessions of TACE post sorafenib^a^	0.9 (0–2)	

a Mean (range).

ECOG, Eastern cooperative oncology group; BCLC, Barcelona clinic liver cancer; TACE, Transarterial chemoembolization.
